# Detection and genotyping of *Helicobacter pylori* in saliva versus stool samples from asymptomatic individuals in Northeastern Thailand reveals intra-host tissue-specific *H. pylori* subtypes

**DOI:** 10.1186/s12866-018-1150-7

**Published:** 2018-01-30

**Authors:** Phattharaphon Wongphutorn, Chariya Chomvarin, Banchob Sripa, Wises Namwat, Kiatichai Faksri

**Affiliations:** 1 Department of Microbiology, Faculty of Medicine, Khon Kaen University, Khon Kaen, 40002 Thailand; 2 Liver Fluke and Cholangiocarcinoma Research Center, Khon Kaen University, Khon Kaen, 40002 Thailand; 3 Department of Pathology, Faculty of Medicine, Khon Kaen University, Khon Kaen, 40002 Thailand; 4 Research and Diagnostic Center for Emerging Infectious Diseases (RCEID), Khon Kaen University, Khon Kaen, 40002 Thailand

**Keywords:** *Helicobacter pylori*, Genetic diversity, Genotyping, Saliva, Stool, *vac*A

## Abstract

**Background:**

Two-thirds of the world’s population is thought to be infected by *Helicobacter pylori*. Although most people infected with *H. pylori* are asymptomatic, this pathogen is associated with several gastric pathologies including cancer. The risk factors for colonization are still unclear and the genetic diversity within individual hosts has never been clearly investigated.

**Result:**

This study determined the prevalence of, and explored risk factors for, *H. pylori* infection directly from paired saliva (*n* = 110) and stool (*n* = 110) samples from asymptomatic persons in Northeast Thailand. Samples were subjected to indirect immunofluorescence assay (IFA), 16S rRNA-based real-time PCR and *vac*A-based semi-nested PCR. Partial *vac*A gene sequences of *H. pylori* were compared between saliva and stool samples.

The overall prevalence of *H. pylori* infection in our asymptomatic study population was 64%. Age, gender, occupation and frequency of brushing teeth were not found to be associated with *H. pylori* colonization. The *vac*A gene was successfully sequenced from both saliva and stool samples of 12 individuals. For seven of these individuals, saliva and stool sequences fell into different clusters on a phylogenetic tree, indicating intra-host genetic variation of *H. pylori.*

**Conclusion:**

This study reports a high prevalence of *H. pylori* infection in asymptomatic persons in this region of Thailand and demonstrates that genotypes (*vac*A gene sequences) of *H. pylori* may differ between the oral cavity and intestinal tract.

**Electronic supplementary material:**

The online version of this article (10.1186/s12866-018-1150-7) contains supplementary material, which is available to authorized users.

## Background

*Helicobacter pylori* infection is responsible for several gastric diseases, especially major ulcers in the stomach and small intestine. Transmission mechanisms of *H. pylori* are still unclear but the fecal-oral route might be the most important [1]. Approximately two-thirds of the world’s population is infected by *H. pylori*, with the highest prevalences (up to 80%) in developing countries [2]. In contrast, prevalences are tending to decline in industrialized countries but are typically in the region of 40% [1, 3]. Although *H. pylori* infection remains asymptomatic in most people, its presence is associated with an increased risk of various gastric pathologies, especially gastric cancer [4]. Moreover, this pathogen might be involved in the pathogenesis of some liver diseases such as gall stone, chronic cholecystitis and cholangiocarcinoma [5–9].

The isolation of *H. pylori* is difficult due to the fastidious nature of the organism. This affects estimates of prevalence: different methods and different types of sample can yield different results [10–12]. Previous estimates of the prevalence of *H. pylori* infection in stool samples of asymptomatic persons have ranged from 6.8% to 73.3% [10–14]. Corresponding values for saliva samples are 45.7%–67.8% [10, 11, 15]. In Thailand, only one study has so far investigated the prevalence of *H. pylori* infection in stool samples of asymptomatic persons [13]. No study has combined several methods of detection to compare the prevalences and genotypes of *H. pylori* infection from paired stool and saliva samples. Although socioeconomic status, especially hygienic conditions during childhood, was suggested to be associated with *H. pylori* infection [1], risk factors for colonization are still unclear.

Each *Helicobacter* species has a preferred niche within the body, facilitating their classification into gastric (localize in gastric mucosal surface) and enterohepatic (localize in ileum, colon and biliary tree) species [1]. *H. pylori* is a gastric species. A few studies have investigated the genetic variation of *H. pylori* among tissue sites based on presence/absence of genetic structure, but used methods with low discriminatory power relative to gene sequence analysis [16, 17]. One study has compared *ureC* sequences of *H. pylori* between saliva samples and gastric biopsies from three patients, but no clear difference was found [18]. There have been no previous studies to investigate intra-host genetic diversity based on gene sequences among different tissues in asymptomatic individuals.

This study aimed to 1) determine the prevalence of *H. pylori* infection in asymptomatic persons in Northeast Thailand using a combination of molecular and immunological methods; 2) investigate the association between the *H. pylori* colonization and demographic-socioeconomic status of the host, and 3) investigate the intra-host genetic diversity of *H. pylori* by comparing strains isolated from saliva and stool samples.

## Methods

### Setting and populations

One-hundred and ten healthy persons (44 males and 66 females) living in Mancha Khiri district (*n* = 80), Khok Pho Chai District (*n* = 10) in Khon Kaen Province and Kamalasai District in Kalasin Province (*n* = 20) of Thailand were recruited into the study during the period March 2014 to December 2015. The participants (i) were between 18 and 60 years of age, (ii) had no previous diagnosis of gastric carcinoma, gastritis, dyspepsia or other gastro-hepatobiliary diseases, (iii) had no history of severe alcohol abuse, (iv) reported no usage of proton pump inhibitor (PPI), bismuth-containing compounds or antibiotics within 4 weeks prior to recruitment.

### Data and specimen collection

Demographic data were collected by questionnaire used previously [19]. Saliva collection was performed according to [20] with slight modification by including post-collection enrichment on Brucella broth. Approximately 3 ml of saliva and 2 g of stool samples were collected from each participant and stored at − 80 ^๐^C. Written ethical consents were received from participants. The study was approved by Institutional Human Ethics Committee of Khon Kaen University (HE571489).

### DNA extraction

DNA was extracted from 1 ml of saliva and 1 g of stool samples using the Puregene DNA Purification System (Gentra System, USA) and QIAamp® Fast DNA Stool Mini Kit (Qiagen, USA), respectively, according to manufacturers’ instructions.

### Semi-nested PCR for detection of *H. pylori*

*vac*A of *H. pylori* was amplified by semi-nested PCR using specific primers, vac F1/F2 and R1 followed by vac F1/F2 and R2 (Table 1). PCR was performed in a total volume of 25 μl containing 500 ng DNA template, 1X PCR buffer (+ 1.5 mM MgCl_2_), 0.4 μM of each primer, 0.5 unit of *Taq* DNA polymerase (RBC bioscience, Taipei, Taiwan) and 0.2 mM dNTP (Amresco, Ohio, USA) using a thermal cycler (C1000™ Thermal Cycler, BioRad). PCR conditions and primer sequences are shown in Table 1. Amplified products were separated by electrophoresis in a 1.5% agarose gel and visualized by staining with ethidium bromide.

**Table 1:** Primer sequences and PCR conditions for molecular detection of *H. pylori*

Genes	Primer names: sequences (5′ > 3′)	Product size (bp)	PCR conditions	Ref.
*vac*A	F1/2: GCATGATTTTGGCACCATTG	429	95 °C 30 s, 52 °C 30 s,	[21]
	R1: TTTTCATATTTAGGGGCAAA		72 °C 45 s (35 cycles)	
	F1/2: GCATGATTTTGGCACCATTG	276	95 °C 30 s, 62 °C 30 s,	
	R2: ATCGCATTGCTCAAGCTCAA		72 °C 45 s (35 cycles)	
16S rRNA	F: CTCATTGCGAAGGCGACCT	139	95 °C 20 s, 58 °C 30 s,	[43]
	R: TCTAATCCTGTTTGCTCCCCA		72 °C 45 s (35 cycles)	

### SYBR-green real-time PCR for detection of *H. pylori*

A portion of the 16S rRNA of *H. pylori* was amplified using a PCR mixture containing 2X of SsoAdvanced™ universal SYBR® Green supermix (BioRad, Canada), 1 μM of each specific primer and 100–500 ng of DNA template. Distilled water was added to produce a final volume of 10 μl and the reaction was run in a CFX96™ Real-Time System. The results were analyzed using CFX Manager Version 3.1 (BioRad, USA). The PCR conditions and primer sequences are shown in Table 1.

### Indirect immunofluorescence assay (IFA) for detection of *H. pylori*

IFA was performed according to the protocol previously described [21]. For saliva, 1 ml of each sample was centrifuged at 13,000 rpm for 10 min and supernatant was discarded and then 1 ml of Brucella broth (CRITERION, USA) was added to the pellet and incubated for 3 days. One ml of suspension was then centrifuged at 13,000 rpm for 5 min and the pellet was washed 3 times using PBS. Then, 30 μl of cell suspension was dropped onto a slide and allowed to dry. Then, 30 μl of primary mouse anti-*H. pylori* IgG (Santa Cruz Biotechnology, USA) was added onto the slide and incubated at 4 °C for at least 1 h. The slide was washed 3 times using PBS, dried and then 30 μl of secondary goat anti-mouse IgG-conjugated fluorescein isothiocyanate (FITC) (Dako®, Denmark) was added and incubated for 1 h. The slide was washed using PBS and dried. Then, 5 μl of mounting solution (PBS and glycerol; 50:50) was added and closed by cover slip. Finally, sample slides were observed for the presence of fluorescent bacteria under a fluorescence microscope (Nikon, Japan).

For stool, 1 ml of Brucella broth was added to 1 g of stool sample and homogenized. The suspension was then centrifuged at 13,000 rpm for 1 min. Supernatant was separated and then 500 μl of Brucella broth was added to the supernatant and incubated for 3 days. One ml of suspension was centrifuged at 13,000 rpm for 5 min and pellet was washed 3 times using PBS. Then, 30 μl of cell suspension was dropped onto a slide, dried and further tested as described above.

### Limits of detection (LODs) of *H. pylori* using molecular methods and the immunological method

Methods for determining LODs in spiked-stool samples were modified from [22]. To prepare spiked-stool samples, bacterial suspension of *H. pylori* was added to produce stool containing from 10^0^ to 10^8^ cells/ml. The crude DNA sample from each dilution was extracted from 1 ml of stool suspension using a commercial DNA extraction kit (QIAamp® Fast DNA Stool Mini Kit; Qiagen, USA) and was used to determine the LODs of semi-nested PCR and real-time PCR. One ml of bacterial suspension from each dilution was inoculated into 0.1 g of stool samples and was used to determine the LOD of IFA.

### *vac*A sequencing and phylogenetic analysis

Amplified products of *vac*A (276 bp) of *H. pylori* from all *vac*A-positive samples based on semi-nested PCR were sequenced by a DNA sequencing service (BIONEER, Korea) in both forward and reverse strands.

Phylogenetic analysis of *H. pylori* strains based on *vac*A was performed based on the maximum likelihood (ML) method in MEGA-7 [23] using the Kimura 2-parameter model of nucleotide substitution and a gamma model of rate heterogeneity (the model giving the best log-likelihood value). The phylogenetic tree was constructed based on 1000 bootstrap replicates.

### Data analysis

Criteria for a true positive result of *H. pylori* detection were modified from [24]: at least two of the following three methods must be positive for a given sample; IFA, semi-nested PCR and SYBR-green real-time PCR. Descriptive statistics were used to describe overall results. Pearson’s chi-squared test was used to analyze the relationship between socioeconomic status, oral hygiene and the presence of *H. pylori* in saliva and stool samples. Odds ratios (ORs) and 95% confidence intervals (CIs) were calculated. Multivariate logistic regression analysis was used to evaluate relationships of risk factors to *H. pylori* infection by adjusting for age, and gender. SPSS version 16 (SPSS Inc., Illinois, USA) was used. A *p*-value <0.05 was considered to be statistically significant.

## Results

### Prevalence and detection of *H. pylori* in 110 healthy, asymptomatic persons in northeast Thailand

**Fig. 1:**
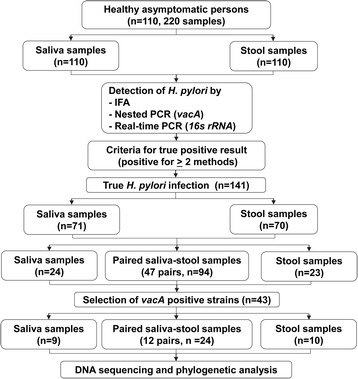
Study population and classification criteria of the tested samples

**Fig. 2:**
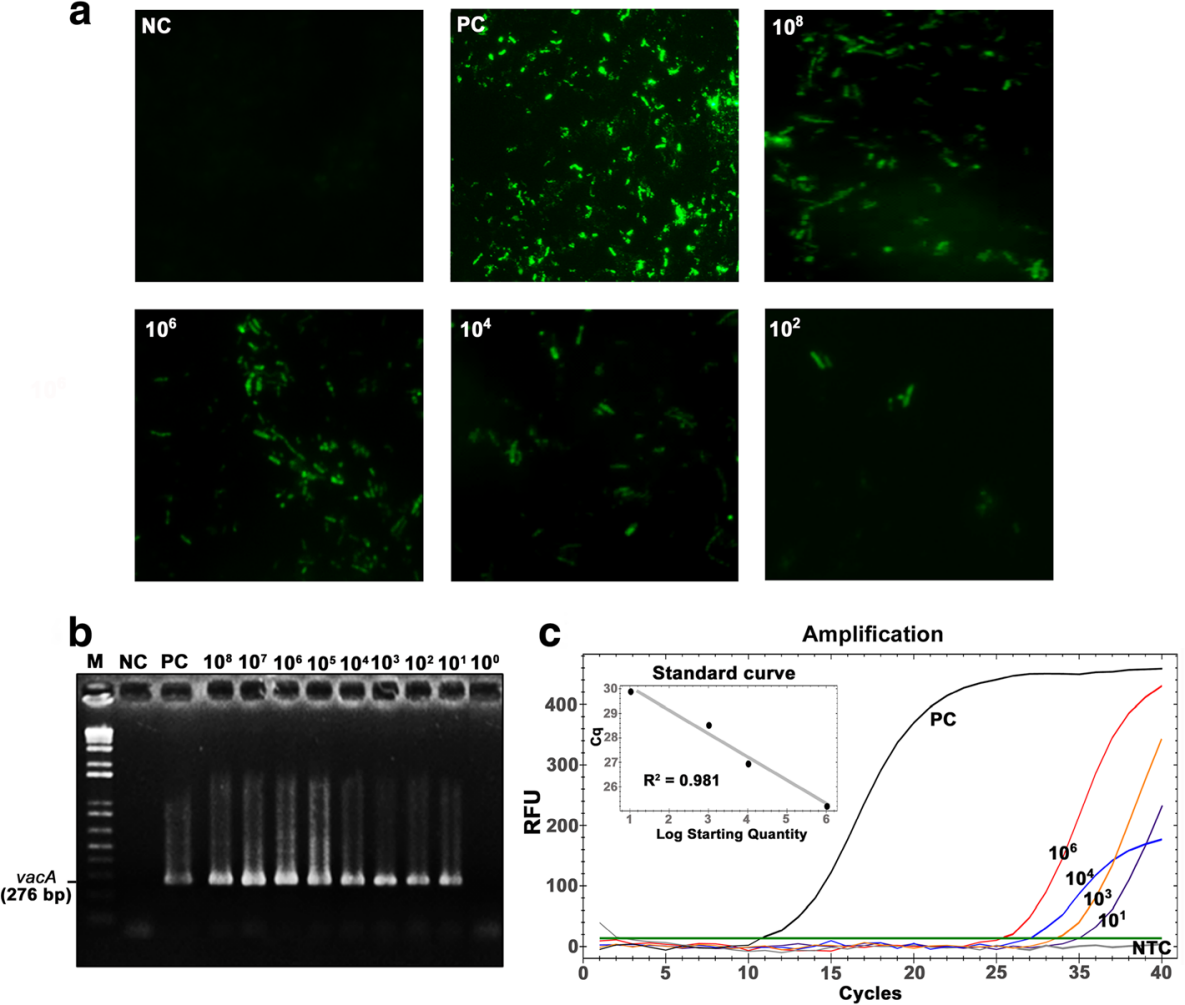
Detection of *H. pylori* directly from stool samples using IFA, semi-nested PCR targeting *vac*A and real-time PCR targeting 16S rRNA. (A) Fluorescent photomicrograph of *H. pylori* in various concentrations (CFUs/0.1 g), NC and PC refers to negative (sterile Brucella broth) and positive control (*H. pylori* DMST 20165 in Brucella broth), respectively. (B) Semi-nested PCR targeting *vac*A in spiked stool samples. Lane M, 1 kb DNA ladder; Lane 1, negative control (PCR reagent without DNA); Lane 2, positive control (DNA sample from *H. pylori* DMST20165); Lanes 3–11, stool samples spiked with various numbers of *H. pylori* cells (10^8^–10^0^ CFUs/0.1 g). (C) Amplification curve of SYBR green real-time PCR targeting 16S rRNA in spiked stool samples. Black line, positive control (DNA sample from *H. pylori* DMST20165); Red line, 10^6^; orange line, 10^4^; blue line, 10^3^; purple line, 10^1^ CFUs/0.1 g; grey line, Non-template control (PCR reagent without DNA)

From 110 saliva samples, 65 (59.1%), 72 (65.5%) and 57 (51.8%) were positive according to semi-nested PCR, real-time PCR and IFA, respectively. Similarly, 50 (45.5%), 80 (72.7%) and 59 (53.6%) of stool samples were positive according to semi-nested PCR, real-time PCR and IFA, respectively. Seventy-one (64.5%) stool and 70 (63.6%) saliva samples were positive for *H. pylori* according to at least two of the methods used (Additional file 1: Table S1). *H. pylori* was detected from both saliva and stool samples in 47 (42.72%) persons (i.e. 94 true-positive samples from these individuals). From among these 141 samples, 70 were randomly selected for *vac*A PCR/sequencing, but sequencing was only successful for 43 samples. Twenty-four of the sequenced samples were matched stool and saliva from each of 12 individuals: saliva samples only were sequenced from a further nine individuals, and stool samples only from a further ten individuals (Fig. 1). There were 98/110 (89.1%) saliva and 91/110 (82.7%) stool samples that were positive to at least one method. In spiked stool samples, the limit of detection (LOD) of semi-nested PCR targeting *vac*A was 10^1^ CFUs/0.1 g. The limit for real-time PCR targeting 16S rRNA gene was also 10^1^ CFUs/0.1 g, and for IFA the limit was 10^2^ CFUs/0.1 g (Fig. 2).

### Risk factors for *H. pylori* colonization in oral cavity and intestinal tract

The risk factors for *H. pylori* colonization were investigated. Most infected participants were female, aged more than 40 and worked in agriculture. There was no significant association between *H. pylori* colonization and risk factors including age, gender, occupation and frequency of brushing teeth (Table 2).

**Table 2:** Risk factors for colonization of *H. pylori* in saliva and stool samples of asymptomatic patients

Risk factors for *H. pylori* colonization	*H. pylori* detection* (n, %)		Crude odds ratios (95% CI)	Adjusted odds ratios*** (95% CI)
Saliva samples	Positive (*n* = 71)	Negative (*n* = 12)		
≥ 40 years	60 (84.5%)	11 (91.7%)	0.50 (0.06–4.24)	0.45 (0.05–3.85
Female gender	41 (57.7%)	8 (66.7%)	0.68 (0.19–2.48)	0.91(0.27–3.10
Agriculturist	62 (87.3%)	11(91.7%)	0.63(0.07–5.45)	0.59 (0.07–5.25)
Teeth brushing ≥ 2-time a day**	69 (97.2%)	11 (91.7%)	3.13 (0.26–37.58)	4.26 (0.29–63.04)
Stool samples	Positive (*n* = 70)	Negative (*n* = 19)		
≥ 40 years	61 (87.1%)	16 (84.2%)	0.80 (0.16–4.04)	0.66 (0.13–3.34)
Female gender	40 (56.3%)	14 (73.7%)	0.48 (0.15–1.47)	0.70 (0.25–1.96)
Agriculturist	64 (91.4%)	15 (78.9%)	2.00 (0.45–8.88)	3.27 (0.77–13.97)
Teeth brushing ≥ 2-time a day**	68 (97.1%)	18 (94.7%)	1.76 (0.15–20.51)	2.62 (0.20–34.82)

*Only true positives (positive according to at least 2 methods) and true negatives (negative by all methods) were included in the analysis

**Teeth brushing once a day was used as reference

***Multivariate analysis was adjusted for age and gender

### Genetic diversity and association of *H. pylori* in saliva and stool samples

**Fig. 3:**
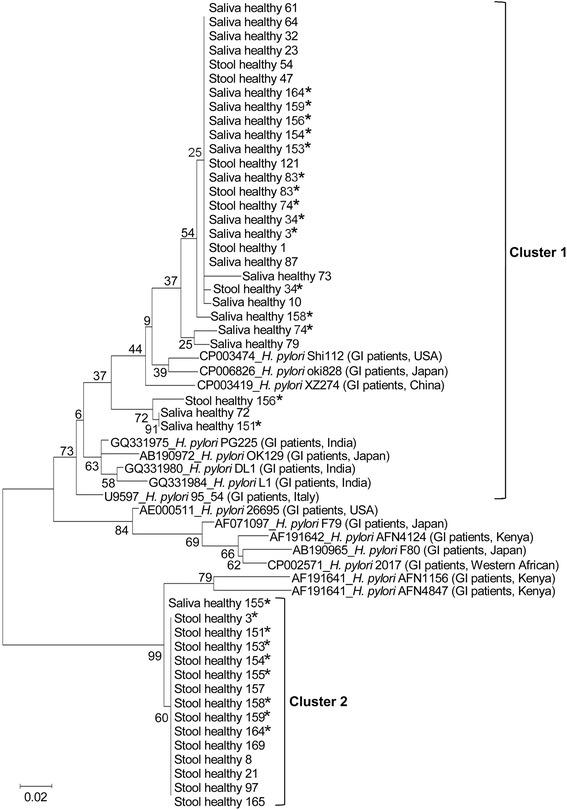
Maximum Likelihood tree of 276-bp *vac*A gene *H. pylori* isolated from saliva and stool samples of asymptomatic individuals from Northeastern Thailand. An asterisk indicates the 12 subjects from which the *vac*A gene in both saliva and stool samples were sequenced. In seven out of 12 pairs (Healthy 3, 151,153, 154, 158, 159 and 164), sequences of *H. pylori* detected from saliva samples were in a separate cluster to those from stool samples

Phylogenetic analysis of *H. pylori* placed sequences of the *vac*A gene into two main groups (Fig. 3). Cluster 1 is a mixed group comprising 8 strains from stool samples, 20 strains from saliva samples and 8 reference strains from different countries. Cluster 2 comprised 14 strains from stool samples and only a single strain from saliva and this proportion is significantly different (*p* = 0.001). Based on the 12 subjects from which both saliva and stool samples were sequenced, 7 pairs were in separate clusters (cluster 1 saliva and cluster 2 for stool samples).

## Discussion

*H. pylori* is a fastidious bacterium that can convert into a viable but non-culturable state, leading to difficulty in identifying its presence [25]. On the other hand, the detection of *H. pylori* using antigen or PCR-based methods directly from clinical samples might be non-specific due to cross reactivity. These limitations could have affected estimates of the prevalence of *H. pylori* infection in previous studies. Detection of several target genes together, such as *ure*A, *glm*M, *ure*C, 16S rRNA, 23S rRNA, *hsp*60, and *vac*A, helps to reduce the number of false positive results, especially for samples other than gastric biopsy specimens [25]. Therefore, we used stringent criteria for detection of *H. pylori* directly from saliva and stool samples based on in-house IFA and molecular methods including semi-nested PCR (*vac*A) and SYBR-green real-time PCR (16S rRNA), the latter modified from [24]. We found that the prevalences of *H. pylori* in asymptomatic healthy persons are comparable between saliva and stool samples in our region at 64%. This number is similar to the prevalences of *H. pylori*, detected based on 16S rRNA, in asymptomatic subjects in India (66.7% in saliva and 77.3% in stool samples) [10]. However, such estimates can vary depending on the region of study and method of detection. For example, one study in India, using PCR targeting *hsp*60, found prevalences of 45.7% in saliva and 42.8% in stools [11]. A study in Nigeria, using PCR targeting *glm*M, estimated a prevalence of only 20.6% [14]. A previous study from Thailand, using detection of 16S rRNA in stool samples, estimated a prevalence of 38.5% [13]. We required positive results from at least two testing methods before accepting that a sample was positive. If we had accepted only a single positive test result, our estimate of prevalence would have been even higher, around 80%. Differences in prevalence estimates between the previous study and ours might reflect a higher current prevalence of *H. pylori* in healthy persons*.* The previous study in India [11] found older age and occupation as cattlemen and agriculturists to be associated with *H. pylori* infection [26]. Factors contributing to the high prevalence we found in our study, such as age and occupation, should be considered.

So far, *vac*A has been detected in all studied *H. pylori* strains [27–30] and hence has been used as a specific, conserved target for detection of this species. Our study showed that combined semi-nested PCR targeting *vac*A and real-time PCR targeting the 16S rRNA gene increased the detection rate, as one target only may occasionally fail to amplify. The IFA technique can detect viable cells of *H. pylori* using specific antibodies against bacterial protein [31]. The LOD of this method in spiked stool samples was 10^2^ cells/0.1 g. Both of the PCR-based methods used in our study had LODs at 10^1^ cells/0.1 g in spiked stool samples. These values are similar to those obtained in a previous study from our group that showed LODs at 10^1^ cells/ml in pure culture and spiked saliva samples [32].

Risk factors for *H. pylori* infection remain unclear. Socioeconomic conditions such as young age, low income and crowded dwellings have been reported as associated with *H. pylori* infection [33]. We found no significant association between *H. pylori* colonization and age, gender, occupation or frequency of brushing teeth. Reported prevalences of *H. pylori* infection were as low as < 5% in saliva and stool samples of children aged < 5 years, but had increased to approximately 58.3% in the age group of 11–16 years and remained static in the range of 56–63% up to the age of 60 years [11]. Perhaps this explains why we failed to demonstrate an association between age and infection: most of our participants were adults. Some previous studies have reported significantly higher prevalences of *H. pylori* in males than in females [34, 35]. Others have found no association with gender [11, 15], a result that agrees with our findings. Higher prevalences have been reported previously in cattlemen and agriculturists, suggesting possible zoonotic transmission [26]. Living in rural areas and exposure to poor hygiene conditions was speculated as a major risk factor [36, 37]. However, despite the fact that the subjects in our study lived in rural areas and most were agriculturists (89.1%), no significant association between occupation and the presence of *H. pylori* in saliva and stool samples was found. Previously, poor oral health and oral diseases have been reported as associated with the infection of *H. pylori* in the oral cavity [38]. Others have found no correlation between the presence of *H. pylori* in the oral cavity and oral hygiene habits (e.g. teeth brushing and the use of mouthwash) [39]. We found no association between frequency of brushing and *H. pylori* colonization.

Momtaz et al. [16] genotyped *vac*A and *cag*A from saliva, gastric biopsies and stool samples and concluded that *H. pylori* was transmitted via the fecal-oral route. We investigated the phylogeny of *H. pylori* based on sequences within the conserved region of the *vac*A gene from saliva and stool samples. We found that 7/12 participants (for whom sequences were obtained both from saliva and stool samples) contained *H. pylori* strains associated with tissue location, i.e. oral strains largely belonged to cluster 1 and stool strains to cluster 2 in the phylogenetic tree. This suggests that particular *H. pylori* strains might have preferred tissue sites for colonization or differential ability to survive within the gastrointestinal tract such as the acidic site inside the stomach. Vacuolating cytotoxin A, encoded by *vac*A, is involved in several processes of pathogenesis including pore formation of the infected cells [40]. A pH-dependent structural change in vacuolating cytotoxin A has been reported [41]. Genetic variants of *vac*A might exhibit differences in toxin activity in the acidic environment of the stomach, influencing bacterial survival. Further study of this possibility is needed.

Previous studies could not identify distinct genetic variants that might be associated with particular sites in the host. This might have been due to their use of methods with low discriminatory power [16, 17] or be a consequence of small sample size [18]. In our study, the majority (7/12) of individuals for which both stool and saliva samples were sequenced contained *H. pylori* strains associated with tissue location, i.e. oral strains largely belonged to cluster 1 and stool strains to cluster 2 in the phylogenetic tree. Based on our observations under the microscope, the sizes of the bacterial cells and colonies are similar between strains isolated from the two sites. Because *H. pylori* is a fastidious organism and difficult to isolate on artificial media [42], we did not perform the phenotypic assay. Among the five individuals for whom *H. pylori* oral (saliva) and intestinal (stool) pairs of sequences fell in the same cluster, four were in cluster 1. Possibly cluster 1 strains are better able to survive in the stomach and/or intestinal tract. Although we found frequent differences between *vac*A genotypes in the oral cavity and intestinal tract samples from the same individual, the number of these paired samples is still low. Additional study with an increased number of paired samples could overcome this limitation. Further investigation of genetic and strain diversity in *H. pylori* and their ability to survive within different host conditions, such as the acidic conditions in the stomach, is clearly warranted.

## Conclusion

In summary, we found that the prevalence of *H. pylori* in the asymptomatic, apparently healthy population is around 64% in both saliva and stool samples. This high prevalence increases the risk of development of associated diseases. Although the fecal-oral route is the major mode of transmission of *H. pylori*, genotypes of the *vac*A gene often differed between oral cavity and intestinal tract samples. Tissue-specific subtypes of *H. pylori* subtypes might be associated with different virulence properties, a phenomenon requiring further investigation.

## Additional file

Additional file 1: Table S1. Detection of *H. pylori* true positive result in saliva and stool samples. (DOCX 12 kb)

## Abbreviations

CFU: Colony forming unit
IFA: Indirect immunofluorescence assay
LODs: Limit of detections
*vac*A: Vacuolating cytotoxin A
