# Supplementary material for: Detection and genotyping of Helicobacter pylori in saliva versus stool samples from asymptomatic individuals in Northeastern Thailand reveals intra-host tissue-specific H. pylori subtypes
Source: BMC Microbiol. 2018 Jan 30;18:10. doi: 10.1186/s12866-018-1150-7 (PMC5789744; doi:10.1186/s12866-018-1150-7)
Supplement: Additional file 1: Table S1. — Detection of H. pylori true positive result in saliva and stool samples. (DOCX 12 kb) [file 12866_2018_1150_MOESM1_ESM.docx]

**Additional file**

**Table S1**.

Detection of *H. pylori* true positive result in saliva and stool samples

| Methods of detection | | | Results | Saliva (n=110) | Stool (n=110) |
| --- | --- | --- | --- | --- | --- |
| IFA | Real-time PCR | Semi-nested PCR |  |  |  |
| + | + | + | TP | 25 | 28 |
| - | + | + | TP | 26 | 16 |
| + | + | - | TP | 11 | 20 |
| + | - | + | TP | 9 | 6 |
| + | - | - | FP | 12 | 5 |
| - | + | - | FP | 10 | 16 |
| - | - | + | FP | 5 | 0 |
| - | - | - | TN | 12 | 19 |
| Total of true positive (%) | | |  | 71 (64.5) | 70 (63.6) |

IFA: immunofluorescence assay, TP: true positive result, FP: false positive result, TN: true negative result. True positive: positive according to at least 2 methods, True negative: negative result from all methods.
